# Investigation of a Gas Hydrate Dissociation-Energy-Based Quick-Freezing Treatment for Sludge Cell Lysis and Dewatering

**DOI:** 10.3390/ijerph16193611

**Published:** 2019-09-26

**Authors:** Woojeong Kim, Hyung Kae Lee, Young-Nam Kwon

**Affiliations:** School of Urban and Environmental Engineering, Ulsan National Institute of Science and Technology (UNIST), Ulsan 44919, Korea; woojeong0549@unist.ac.kr (W.K.); sosky@unist.ac.kr (H.K.L.)

**Keywords:** gas hydrate dissociation energy, quick freezing, sewage sludge, cell lysis, dehydration

## Abstract

A gas Hydrate dissociation-energy-based Quick-Freezing treatment (HbQF) was applied for sewage sludge cell rupture and dewatering. Carbon dioxide (CO_2_) and water (H_2_O) molecules in sewage create CO_2_ gas hydrates, and subsequently the sludge rapidly freezes by releasing the applied pressure. Cell rupture was observed through a viability evaluation and leachate analysis. The decreased ratios of live cell to dead cells, increased osmotic pressure, and increased conductivity showed cell lysis and release of electrolytes via HbQF. The change in physicochemical properties of the samples resulting from HbQF was investigated via zeta potential measurement, rheological analysis, and particle size measurement. The HbQF treatment could not reduce the sludge water content when combined with membrane-based filtration post-treatment because of the pore blocking of fractured and lysed cells; however, it could achieve sludge microbial cell rupture, disinfection, and floc disintegration, causing enhanced reduction of water content and enhanced dewatering capability via a sedimentation post process. Furthermore, the organic-rich materials released by the cell rupture, investigated via the analysis of protein, polysaccharide, total organic carbon, and total nitrogen, may be returned to a biological treatment system or (an) aerobic digester to increase treatment efficiency.

## 1. Introduction

As industry develops and population grows, new synthetic pollutants in wastewater are emerging and pollution levels are increasing, and thus, purification by nature is sometimes not sufficient. Various methods including primary, secondary, and advanced treatments have been studied and proposed to artificially treat contaminated wastewater. Each uses physical, biological, or chemical treatment, and during the treatment a large amount of sludge, which is a mixture of highly concentrated organic, inorganic, and microbial waste, is produced. Its high organic and toxic concentration results in several environmental problems. In South Korea, wastewater treatment plants released 32% more sludge during 2013 (10,946 tons/day) compared to that during 2008 (7446 tons/day) [1]. The sludge has been disposed of by ocean dumping, landfilling, and incineration. Landfilling is a process of removing sludge by burying it underground. This method is the simplest and easiest sludge treatment method, but it requires a large area and the highly polluted sludge leachate can pollute the ground and groundwater. Incineration burns the sludge, requiring a smaller area. However, it has very low energy efficiency because of the high water content of the sludge and high energy requirement to burn with water. Compared to the spatial limitation of landfills and high energy requirements of incineration, ocean dumping has been more commonly applied because of its convenience. During 2010, approximately 5.34 million cubic meters of waste and sewage from livestock farms, leftover food, and urban areas were dumped [2] into the ocean. However, since the 1996 protocol, the revised version of the London Convention on the Prevention of Marine Pollution by Dumping of Wastes and Other Matter (1975), ocean dumping has been gradually banned.

Many research studies have been developed to replace ocean dumping. Physical, electrical, and chemical treatments are used along with landfills and incineration. Physical sludge treatment dewaters sludge by physical force, including coagulation-assisted settlement [3,4], centrifugation [5,6], and filter pressing [5,6]. These treatments can decrease the sludge water content by reducing the free water while leaving intercellular water within cells, and thus the methods are limited to a reduction to a certain level or less. Electrical treatment is a sludge dewatering method to disintegrate sludge particles by electrical oxidation. Electrolysis, among the electrical sludge pretreatments, is a method in which electricity is charged to two electrolytes in the sludge, oxidation occurs, and subsequently the oxidation disintegrates and dewaters the sludge [7]. However, as electrolysis needs considerable energy to produce electricity, it has a low energy efficiency. Chemical sludge treatment includes coagulation and acid/base treatment [8,9,10]. During coagulation, cationic ions connect with a negatively charged sludge surface, coagulating the sludge and releasing free and interstitial water. Acid treatment makes protons to neutralize the negatively charged sludge surface. The neutralized sludge surface can coagulate and dewater the sludge. However, the remaining chemicals and acidic pH in the sludge cause environmental pollution when they are released without further treatment. Chemical treatment based on oxidation uses radical chemicals to attack and dewater the sludge. Fenton oxidation [11], ultrasonication [12], ozone [13], and enzymatic treatments [14] are chemical treatments for sludge dewatering via oxidation. In Fenton oxidation, the reaction between Fe^2+^ and H_2_O_2_ forms an OH radical that oxidizes organic materials in sludge to increase the dewaterability. Ultrasonication treatment is the method in which, after a radical is formed by cavitation, it disintegrates the organic materials in the sludge. Ozone treatment applies ozone, a highly oxidizing agent, to oxidize organic materials. Enzymatic treatment uses an enzyme to oxidize organic materials in sludge and help dewater the sludge. However, as oxidization occurs under an acidic condition, its product, acidic sludge, needs post treatment for neutralizing its pH and making the whole treatment cost expensive.

The sludge-freezing method is one of the physical dewatering treatments that result in the inner and outer water of the sludge cells freezing at a low temperature, breaking cells and disintegrating the sludge to drain the internal water along with outer water. In sludge, water distribution [15,16,17,18] can be classified as free, interstitial, bound, and inner. Free water is the water out of the sludge flocs and has no interaction with the sludge flocs. Interstitial water is water trapped between sludge flocs. Bound water is attached onto sludge flocs. Inner water is the intercellular water of microbes in sludge. The formed ice in each part might aid in sludge dewatering via three mechanisms as follows [19]: (1) compaction of sludge by ice formation to release interstitial water, (2) enhanced osmotic pressure of a brine solution to extract water from sludge cells, or (3) cell rupture by the expansion of frozen sludge cells to release the inner water of microbes in sludge. The gas hydrate dissociation-energy-based quick-freezing method (HbQF) is an energy-efficient freezing method. Its driving force for freezing is an endothermic process of gas hydrate dissociation caused by a pressure drop. S.W. Han et al. (2016) [20] utilized gas hydrate dissociation to rapidly freeze sea water and the formed ice was separated from brines. After gas hydrate-induced ice desalination, the brackish water reverse osmosis (BWRO) process showed ~14 LMH (Liters per square Meter per Hour) and approximately 99% rejection except for boron at a fairly low applied pressure. Compared to the general freezing method, the HbQF method needs less energy and is quick because of the utilization of the gas hydrate dissociation energy. In addition, the method uses partial gas hydrate formation, unlike the general freezing method requiring ice formation of the whole sample. The HbQF method requires a less harsh temperature condition than that of the general freezing method. In this study, with modification of the gas hydrate-induced ice desalination, HbQF treatment was developed and applied to sludge cell lysis and dewatering. Sewage sludge samples were quick-frozen under various conditions to evaluate the feasibility of HbQF in the field of sludge dewatering. To the best knowledge of the authors, this is the first research applied to cell lysis through HbQF and subsequent microbial deactivation/dewatering of wastewater sludge.

## 2. Materials and Methods

### 2.1. Preparation of the Sewage Sludge

**Figure 1:**
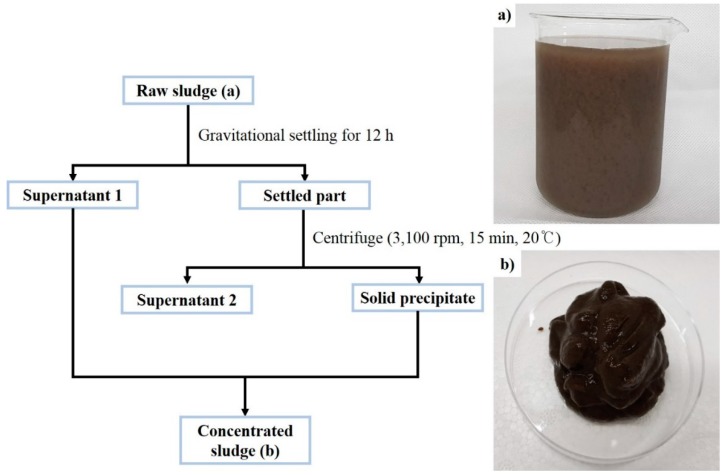
Scheme for preparation of the sewage sludge sample: (**a**) raw activated sewage sludge; (**b**) concentrated sludge.

**Figure 2:**
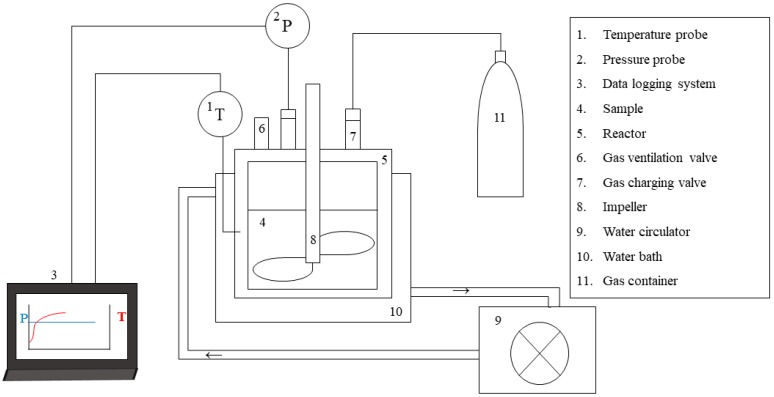
Diagram of the reaction system for HbQF.

Activated sewage sludge (raw sludge (a) shown in Figure 1) was sampled from a wastewater treatment plant in Ulsan, South Korea. To prevent microbial growth and sludge degeneration, the raw solution of the sewage sludge was stored at 4 (±1) °C and experiments/analyses were conducted within 3 weeks of sampling. The raw solution had a high water content, requiring considerable energy and a long time to freeze. The water content of each sludge sample was different depending on the sampling date; thus, the water content of the sludge samples used in these experiments was controlled. The sludge solution was gravitationally settled for 12 h at 4 °C, and then the settled portion was separated by draining the supernatant. The gravitationally settled sediment was, furthermore, centrifuged (3100 rpm, 15 min, 20℃) for thickening. The centrifugation process was completed using a High Speed Refrigerated Centrifuge (VS-24SMTi, VISION SCIENTIFIC CO. LTD, Daejeon, Korea) at 20 °C. The thickened precipitate after centrifugation was separated by draining the supernatant. Finally, the sludge samples used in the HbQF study were prepared by mixing the thickened precipitate and supernatant 1 as shown in Figure 1 at a ratio of 5:1. The sludge mixture (concentrated sludge (b)) as shown in Figure 2 was tested within 24 h.

### 2.2. Gas Hydrate Dissociation-Energy-Based Quick-Freezing Process (HbQF)

Carbon dioxide (CO_2(g)_, Purity 99.999%, Korea SEM, Yangsan, Korea) and nitrogen (N_2(g)_, Purity 99.999%, Korea SEM) were used as guest molecules for the gas hydrate formation in the sewage sludge.

HbQF experiments were conducted using the system shown in Figure 2. The system consisted of a reactor, temperature control unit, and data logging unit. The reactor was composed of a stainless-steel cylinder (diameter of 100 mm and height of 150 mm) and stainless-steel cover. The effective capacity for the reactor was 1.1 L. Two polycarbonate windows were located on the side of the cylinder for observation inside. The temperature of the sludge sample in the reactor was monitored in real time using a temperature probe (SD-560 RS485, omega, Stamford, CT, USA). An impeller was sufficiently large to mix the sludge samples in the reactor. The pressure was controlled through gas injection and release using two valves on the cover. A pressure probe (industrial pressure transmitter A-10, WIKA, Klingenberg, Germany) was on the cover to monitor the pressure inside the reactor. The temperature control unit had a circulating chiller (RW-1025G, Jeio-tech, Daejeon, Korea) and water bath which was transparent for observation. The coolant used in this study was a mixture of water and ethylene glycol to prevent freezing during the experiment. The data logging unit collected real-time data of the temperature and pressure and saved it in a computer.

The HbQF treatment was operated as follows: At first, 500 g of the sludge sample (concentrated sludge (b) as shown in Figure 1) was placed in the reactor. The reactor containing the sludge sample was cooled to 0.2 (±0.1) °C with mixing. After the sludge sample stabilized at 0.2 (±0.1) °C, a guest molecule was purged into the reactor at 1 (±0.3) bar for 10 min to replace the atmospheric gas. After the temperature stabilized at 0.2 (±0.1) °C again, the sludge sample was pressurized to a setting pressure with the guest molecules. After the pressure was maintained for 4 h, the reactor was depressurized to atmospheric pressure for a short time. The sample was separated from the reactor and stored in a refrigerator to melt at 4 °C. The reaction conditions are described in Table 1.

### 2.3. Analysis of Leachate and Sludge

#### 2.3.1. Effect of HbQF on Leachate Characteristics and Cell Viability

Sewage sludge is a mixture of numerous materials including organic, inorganic, and microbes [21]. When a cell is destroyed, intracellular electrolytes and organic materials are released. Therefore, an increase in the amount of soluble organic material and electrolytes means there is cell lysis and sludge disintegration. During this experiment, the release of organic materials and electrolytes by HbQF was investigated.

The leachate of the control and HbQF-treated sewage sludge samples was obtained via centrifugation (20 °C, 5000× *g*, 30 min) using a centrifuge (VS-24SMTi, VISION SCIENTIFIC CO. LTD, Daejeon, Korea) followed by filtration of the supernatant using a 0.8 μm cellulose acetate filter (EW-81054-40, Advantec, Tokyo, Japan). Zeta potential, conductivity, and osmotic pressure of the leachate were measured using a Zetasizer (Marvern Instrument Ltd., Malvern, UK), conductivity meter (ULTRAMETER II™ 6P, Myron L^®^ Company, Carlsbad, CA, USA), and vapor pressure osmometer (Model 5600, ELITechGroup, South Logan, UT, USA), respectively.

Cell lysis resulting from HbQF can deactivate the organisms present in sewage sludge and can decrease waterborne disease. A live/dead bacterial staining kit (L7012, Invitrogen^TM^, Waltham, MA, USA) was used for staining live or dead microbes in the sludge sample. A stained sludge sample was observed using confocal laser scanning microscopy (FV1000, OLYMPUS, Tokyo, Japan) and counted by a software (Imaris, Andor Technology Ltd., London, UK). By counting the stained live and dead particles, the disinfection effect was evaluated.

#### 2.3.2. Effect of HbQF on the Rheology and Particle Size Distribution of the Sewage Sludge

As HbQF causes sludge disintegration and subsequent dewatering, the treatment changes the physical characteristics such as the viscosity of a sludge sample. The thixotropic loop and viscosity analysis were measured using a rheometer (Haake MARS Ⅲ—ORM Package, Thermoelectron, Waltham, MA, USA) with a PP35 Ti sensor (with a cone diameter of 34.004 mm). The condition for the measurements followed the method described by J. Liu et al. [17]. Approximately 1 mL of sludge sample was placed on the plate. Shear stress increased from 0.01 s^−1^ to 1000 s^−1^ to destroy the ordered structure, and shear stress was maintained at the maximum level (1000 s^−1^) for 30 s. Then, shear stress was decreased from 1000 s^−1^ to 0.01 s^−1^ for the recovery of the ordered structure. During measurement, real-time shear stress and shear rate were measured and the viscosity was calculated.

Particle size distribution analysis was conducted in both the μm and nm ranges. For measurement in the μm range, the sludge sample was measured using a particle size analyzer (Mastersizer 3000, Malvern Instrument, Malvern, UK). Its measurement range was from 0.01 μm to 3500 μm. A filtrate was separated from the sludge sample via centrifugation (5000× *g*, 30 min, 20 °C) and subsequent filtration using an 0.8-μm filter. The filtrate was investigated using a zeta-potential analyzer (Nano ZS90, Malvern Instrument, Malvern, UK) for measurement in the nm range (from 0.4–10,000 nm).

#### 2.3.3. Effect of HbQF on Sewage Sludge Water Content

Water content of the sewage sludge was evaluated using three methods as follows: capillary suction time (CST), time to filter (TTF), and settlement. The CST and TTF tests are evaluation methods of sludge dewaterability using a membrane, but the settlement test uses naturally occurring gravitational separation. CST is the time needed for the leachate to horizontally permeate through a certain distance of a filter. Each 5 g of the sludge samples was positioned in the middle of a circular membrane and the time needed for the leachate to travel a certain distance was measured. A filtration test was conducted to understand the effect of the HbQF treatment on sludge dewatering via physical pressurization. Physical pressurization is a basic sludge treatment because of its ease and cost efficiency. A filtration test was conducted using the dead-end filtration system based on Guo, S., et al. [15]. Approximately 20 g of sample was loaded on the filter and pressurized using 1 (±0.2) bar of N_2_ gas. During the filtering test, the weight of the filtrate was recorded every 5 min. TTF is the time at which the weight of the filtrate reaches one-half the weight of the initial sample. For a settlement test, each sample with a mass of ~500 g was placed in a conical glass container and the samples gravitationally settled at room temperature. Images showing the boundary between the solid and liquid parts were compared, and the volume of the liquid portion was measured.

#### 2.3.4. Resistance Analysis

To investigate the effect of the sludge on the filtering test, resistance by various factors [22] was analyzed. It was assumed that the total resistance during the filtration could be divided into three resistances as follows: resistance by membrane, resistance by sludge cake on the membrane surface, and resistance by pore blocking (Equation (1)). A dead-end filtration system was used, and the weight of the filtrate (permeate) was recorded every 10 s. Resistance (R) was calculated using Equation (2) as follows:

(1) $$R_{t}=R_{m}+R_{c}+R_{p}$$

(2) $$R=\frac{\mathrm{Applied}\;\mathrm{pressure}}{\mathrm{Solution}\;\mathrm{viscosity}\times \mathrm{Flux}}$$

First, a filter composed of cellulose ester with a nominal pore size of 0.2 μm (AD.A300A047A, Advantec®, Tokyo, Japan) was submerged in deionized (DI) water for 24 h to fully dehydrate it. A filter was installed in the equipment, and filtration was conducted using DI water at 1 (±0.2) bar of nitrogen gas. Because only pure water passes through the semipermeable membrane, the resistance determining the water flux was a result of the resistance of the membrane itself ($R_{m}$). Then, the sludge sample was placed on the filter and pressurized using 1 (±0.2) bar of nitrogen gas for 3.5 h. After finishing filtration with the sludge sample, DI filtration was again conducted using 1 (±0.2) bar of nitrogen gas. After the sludge filtration was completed, the filter had sludge cake on the membrane surface and fine particles blocking the membrane pores. Therefore, the resistance during the second DI filtration was because of the total resistance ($R_{t}$) combining membrane ($R_{m}$), sludge cake ($R_{c}$) and pore blocking ($R_{p}$) resistance. Then, the filter was washed to remove the cake layer from the membrane surface by shaking with 150 mL of DI water at 100 rpm for 5 min using a shaker (NB-101MT, N-BIOTEK, Bucheon, Korea). After washing the filter, DI filtration was again conducted using 1 (±0.2) bar of nitrogen gas. After washing and filtration with DI, the sludge cake was removed from the surface. Resistance during the third DI filtration was because of the resistances of the membrane and pore blocking ($R_{m}+R_{p}$). $R_{p}$ was calculated by subtracting $R_{m}$ from the resistance from the third DI filtration. Then, resistance by sludge cake ($R_{c}$) was calculated by the difference between the resistance from the second and third DI filtrations.

### 2.4. Release of Organic Materials by HbQF

**Figure 3:**
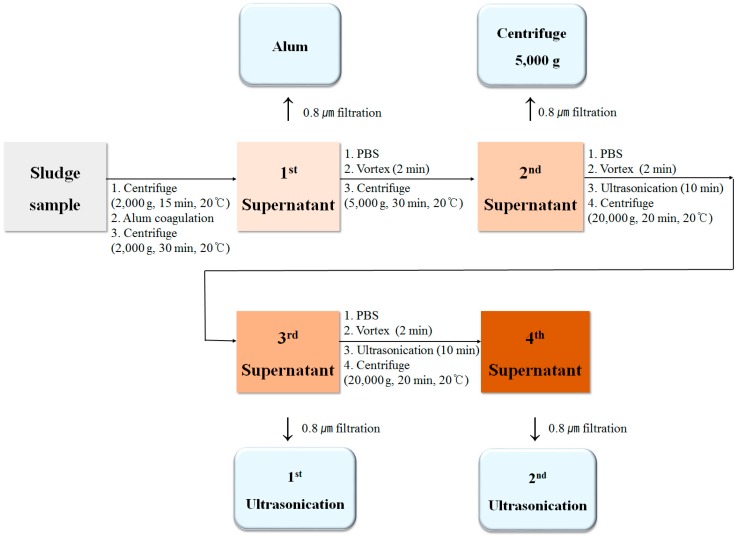
Strength-based separation of organic materials from the sewage sludge.

Organic materials were separated in the sequence from the sludge sample (Figure 3) for the evaluation of the amount of loosely bonded or strongly bonded organic materials.

(a) Step 1: After 30 g of a sludge sample was centrifuged (2000× *g*, 15 min, 20 °C), 0.2 g of aluminum sulfate (Al_2_(SO_4_)_3_, 98%, Sigma-Aldrich, St. Louis, MO, USA) was added, and the mixture was centrifuged (2000× *g*, 30 min, 20 °C). The supernatant was separated and filtered using a 0.45-μm polytetrafluorethylene-H (PTFE-H) syringe filter (SH25P045N, Hyundai micro CO. LTD, Seoul, Korea). The filtrate was termed Alum.
(b) Step 2: Adding phosphate buffered saline (PBS; pH 7.4) into the remaining pellet (from Step 1) to 30 g, the mixture was mixed using a vortex. Then, it was centrifuged (5000× *g*, 30 min, 20 °C). The supernatant was separated and filtered using a 0.45-μm syringe filter. The filtrate was termed Centrifuge 5000× *g*.
(c) Step 3: Adding PBS into the remaining pellet (from Step 2) to 30 g, the mixture was mixed using the vortex mixer. Then, it was treated via Ultrasonication (288,000 W, 10 min) within ice to prevent the sludge heating. The treated sample was centrifuged (20,000× *g*, 20 min, 20 °C) and filtered using a 0.45-μm syringe filter. The filtrate was termed 1st Ultrasonication.
(d) Step 4: Using the remaining pellet from Step 3, Step 3 was repeated. The supernatant was termed 2nd Ultrasonication.

The organic material released in each sample was investigated via three methods/measurements as follows: (1) the phenol-sulfuric acid method [23] with glucose (D(+)-Glucose, anhydrous, 98%, Samchun Pure Chemical) as a standard for the determination of the polysaccharide concentration, (2) the Lowry method [24] (Total Protein Kit, Micro Lowry, Peterson’s Modification (TP0300, Sigma, St. Louis, MO, USA)) with bovine serum albumin (BSA) as a standard for protein concentration, and (3) a total organic carbon and nitrogen (TOCN) measurement using total organic carbon analyzer (TOC-VCPH, Shimadzu Scientific Instruments, Columbia, MD, USA).

## 3. Results and Discussion

A gas hydrate is a substance in which water molecules form a crystal structure around a guest molecule, such that, during its formation, the mobility of water molecules is reduced and the energy is released from the molecules to the system. The process of gas hydrate formation is an exothermic process and thus the temperature in the system increases during the reaction. However, the dissociation of a gas hydrate absorbs the surrounding energy because the molecules in the crystal form must mobilize to collapse the hydrate structure. The dissociation process of a gas hydrate is an endothermic process, and thus the temperature in the system decreases. The formation and dissociation of a gas hydrate can be indirectly observed through a temperature change in the system.

**Figure 4:**
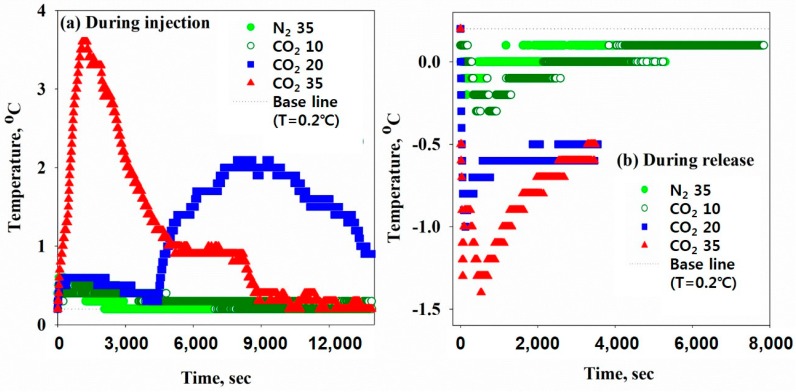
Temperature change of sludge samples in a reactor (**a**) during the injection of the guest gas molecules (CO_2_ and N_2_) at various applied pressures and (**b**) during the subsequent release of the pressurized gas.

Figure 4a shows the temperature increase during the process of pressurizing carbon dioxide or nitrogen gas in a reactor filled with a sludge sample. As the gas was charged in the reactor, the temperature initially increased and then decreased to 0.2 °C, which is the temperature regulated by a connected chiller. The temperature increase of a sludge sample that occurs during the dissolution of a gas in a constant volume of a reactor under applied pressure is determined by three factors: the Joule-Thomson effect and exothermic reactions of both gas dissolution and hydrate formation. When the CO_2_ guest molecules were injected and pressurized into the reactor to maintain 10 bar at 150 rpm, the temperature increased by 0.3 °C. Gas hydrate formation was not observed under this condition, and thus the temperature increase was because of the Joule-Thomson effect and dissolution of CO_2_ (carbonation, CO_2(g) in sludge_ ⇿ CO_2(aq) in sludge_) into water. The elevated temperatures for the CO_2_ at 20 bar and 35 bar were 1.9 °C and 3.4 °C, respectively. In addition to the Joule-Thomson effect and CO_2_ dissolution heat, an exothermic reaction caused by hydrate formation (CO_2(g) in sludge_ + nH_2_O _(__ℓ)_ ⇿ CO_2_·nH_2_O _(s) in sludge_) additionally influenced the temperature increase at pressures above 20 bar. At 20 bar, the hydrate formed after ~4430 s. However, at 35 bar, the hydrate formed immediately after injection of the guest molecule and thus the temperature rapidly increased at the starting point. The higher the applied pressure, the higher the number and density of hydrates produced. Under the same experimental condition (35 bar), the N_2_ and CO_2_ showed different results because of the low solubility of the guest molecule N_2_ (the solubility of CO_2_ and N_2_ in water is 1450 and 17.5 ppm (parts per million) at 25 °C, respectively [25]) and subsequently no gas hydrate formed in the N_2_ reactor. Therefore, the temperature change caused by N_2_ at 35 bar showed similar behavior as the change caused by CO_2_ at 10 bar.

**Figure 5:**
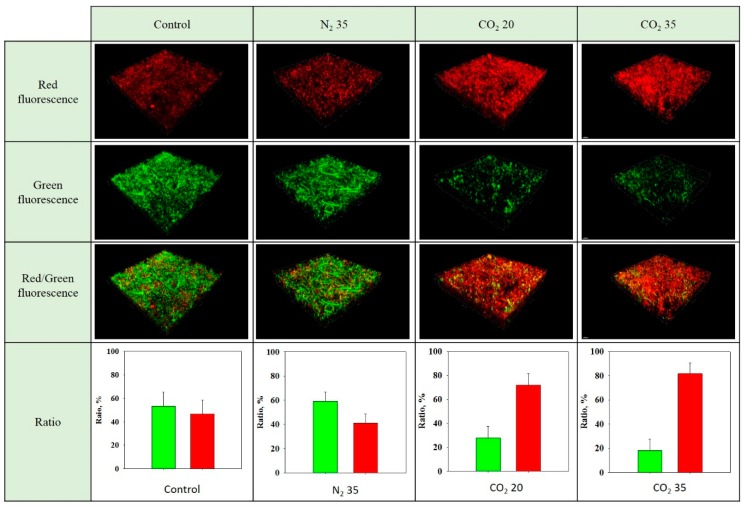
CLSM images of sludge stained by a live/dead *Baclight*^TM^ bacterial staining kit (Invitrogen^TM^, Waltham, MA, USA) and the ratio of green- and red-colored portions in the sludge through the image analysis.

When the valves on the hydrate reactor were opened to release the pressurized guest molecules into the atmosphere, the temperature of the sample decreased and then increased to 0.2 °C (Figure 5b). The reduced temperature in the N_2_ 35 bar case was as small as 0.4 °C. The solubility of the nitrogen gas is much smaller than of carbon dioxide and no hydrates formed under this condition, and therefore, the decrease in temperature was because of the Joule-Thomson effect caused by the gas expansion and endothermic degassing of nitrogen gas from the sludge sample. The reduced temperature in the case of CO_2_ 10 bar was similar to that for the N_2_ 35 bar case. At both N_2_ 35 bar and CO_2_ 10 bar, freezing of the sludge was not observed during degassing in the reactor. However, when the pressure of the CO_2_ was increased to more than 20 bar and then released to atmospheric pressure, quick freezing of the sludge sample was observed. The reduced temperature in the case of CO_2_ at 20 bar was 1.2 °C. The temperature decrease of the sludge sample when the gas hydrate was produced (CO_2_ 20 bar) was greater than that when the gas hydrate was not produced (N_2_ 35 bar and CO_2_ 10 bar). The reduced temperature for the CO_2_ 35 bar was 1.6 °C, and the temperature decrease at 35 bar was more significant than that at CO_2_ 20 bar because more energy was extracted from the dissociation of a greater number of hydrates produced at the higher pressure. S. Han et al. [20] compared the endothermic heats caused by the Joule-Thomson effect, decarbonation of CO_2,_ and dissociation of CO_2_ hydrate using a micro-differential scanning calorimeter (μ-DSC) under the condition of 20 bar and 275.15 K, and showed the heat involved in the decarbonation and CO_2_ hydrate dissociation was 34.48 J/g and 500.13 J/g, respectively, and the heat via the Joule-Thomson effect was negligible. The results of the μ-DSC agree well with the results using the sludge samples in this study.

### 3.1. Effect of HbQF on Sewage Sludge and Leachate Characteristics

#### 3.1.1. Inactivation of Microbial Cells and Cell Lysis in a Sludge Sample

Sewage sludge is composed of many types of microorganisms that are composed of cells containing more than 70% water. Mechanical dewatering methods can remove free water in sludge and some interstitial water captured in the spaces between sludge flocs, but have difficulty in removing the inner water of the sludge cells. Traditional methods have limitations in reducing sludge water content below some point. The extraction of intercellular water within a microorganism through microbial rupture along with breakage of sludge flocs can significantly reduce the sludge water content. The effect of the rapid freezing method, using the endothermic reaction of the CO_2_ gas hydrate dissociation, applied to sludge cell lysis on the viability of microorganisms in sludge was investigated and is shown in Figure 5. The viability was evaluated using a live/dead *Baclight*™ bacterial assay kit (Invitrogen^TM^, Waltham, MA, USA), consisting of SYTO-9 and propidium iodide (PI). SYTO-9 is a membrane-permeable dye and thus passes through live cell membranes to stain nucleic acid and show green fluorescence. However, PI is membrane-impermeable and is therefore blocked by healthy cells, staining the nucleic acid of damaged microbial cells a red fluorescence because PI has stronger affinity for nucleic acid in the presence of both stains. The stained sludge was observed using Controlled Low Strength Materials (CLSMs), in which one can show fluorescence-stained particles in a three-dimensional (3D) image.

In the control sample, the green portion (53% ± 12%) was greater than the red portion (45% ± 12%). The dead cells in the control sample were likely a result of the dead microorganisms in the sludge at the sampling time, the microorganisms that reached the end of their life during sample storage, or the death of some microorganisms during the centrifugation process applied to separate the sludge solids and supernatant during sample preparation. In the case of N_2_ at 35 bar, the green color (59% ± 8%) was more intense than the red color (41% ± 8%), implying that intact cells were more common than the damaged cells. The mechanical stirring, pressure swing, and CO_2_ dissolution/decarbonation did not seem to significantly damage the microbial cells in the sludge. In the sludge samples treated under CO_2_ 20 bar condition, the red color was more intense than green color, indicating intact cells were less common than the damaged cells. The samples that underwent hydrate dissociation and the subsequent quick-freezing process showed a greatly reduced microorganism viability in the sludge (28% ± 10% for CO_2_ at 20 bar and 18% ± 10% for CO_2_ at 35 bar, respectively). This suggests that the quick-freezing method based on the dissociation of the CO_2_ gas hydrate is effective in deactivating microorganisms in the sludge sample.

**Figure 6:**
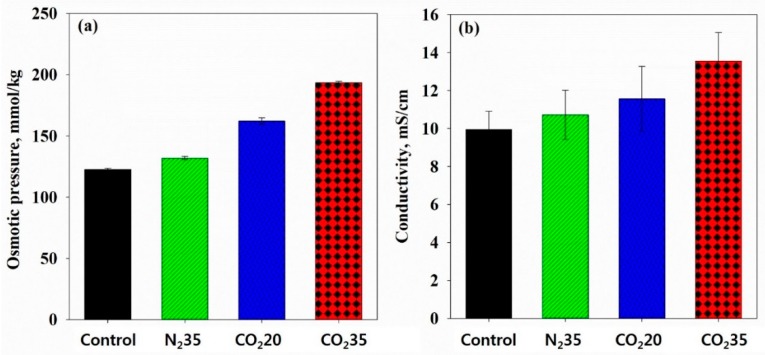
(**a**) Osmotic pressure and (**b**) conductivity of the filtrate separated from the sludge samples.

The release of the electrolytes and small organic materials from the microbial cell lysis was investigated by the change in the osmotic pressure and conductivity in the supernatant of the HbQF-treated sludge samples. The osmotic pressure of the control and N_2_ at 35 bar samples was 122.8 (±0.96) mmol/kg and 132.0 (±1.41) mmol/kg, respectively, as shown in Figure 6. The leakage of the intercellular materials induced by stirring, the pressure swing, and CO_2_ dissociation slightly increased the osmotic pressure of the supernatant. The osmotic pressure of the CO_2_ at 20 bar and CO_2_ at 35 bar samples was 162.3 (±2.63) mmol/kg and 193.5 (±1.29) mmol/kg, respectively, much higher than the control or the N_2_ at 35 bar samples. This shows HbQF caused more severe sludge disintegration. The conductivity of the control and N_2_ at 35 bar samples was as low as 9.836 (±0.9141) mS/cm and 10.571 (±1.2353) mS/cm, respectively; however, the conductivity of the CO_2_ at 20 bar and CO_2_ at 35 bar samples was 11.390 (±1.6458) mS/cm and 13.388 (±1.4177) mS/cm, respectively. The correlation coefficient between the osmotic pressure and conductivity was 0.98. From analysis of the supernatants, it was determined that the electrolyte and/or organic materials released via cell lysis resulted in the supernatant having a higher osmotic pressure and conductivity.

During the decompression process after the formation of the CO_2_ hydrate, the endothermic reaction caused by the gas hydrate dissociation led the water to freeze, and ice was generated both inside and outside of the microbial cells. The cell lysis and dehydration via the HbQF treatment might have occurred because of four reasons: (1) When the ice formed outside the microbial cells, the aqueous solution around the cells became highly concentrated because of the ice formation with pure water, and thus the cells were exposed to the brine solution. Then, the water inside the cell was extracted toward the outside because of the osmotic gradient across the cell membrane and the cell dehydrated. (2) When the ice formed on the surface of the cells, the ice absorbed water from cells for its growth, dehydrating the cell. (3) When the ice formed inside the cell, the inner cell volume suddenly increased and the cell membranes ruptured because water expanded the volume by 9% when freezing. Then, the cell released the inner water. (4) The friction between the solid hydrate/ice and the sludge cells ruptured some cells.

Sludge has charges on the surface, and the surface charge affects sludge flocculation and disintegration. In this study, the surface charge of the sludge particles was measured by the zeta potential (Table 2). It can be measured based on the electrophoretic mobility in the electric field. If the surface charge of particles has a different value, the particles have a different velocity in the electric field and the surface charge can be calculated from its velocity. The zeta potential of the control was −11.12 (±0.743) mV. Because of the ionization of the anionic functional group in the organic materials, the sludge had a negative surface charge [26]. The surface charge of the N_2_ at 35 bar and CO_2_ at 10 bar samples were more negative (N_2_ at 35 bar: −15.40 (±0.400) mV and CO_2_ at 10 bar: −15.30 (±1.916) mV, respectively) than the control. Stirring, the pressure shift, and gas dissolution might disintegrate the sludge and slightly release organic materials. The surface charge of the CO_2_ at 20 bar and CO_2_ at 35 bar samples was more negative (−16.30 (±0.833) mV and −21.00 (±2.258) mV, respectively). Because more negatively charged sludge flocs enhance the electrostatic repulsion force, sludge flocs can be easily dispersed [27]. In this study, HbQF resulted in a more negative sludge surface charge. As the sludge surface charge becomes more negative, the particles attempt to separate and the interstitial water can be easily released.

#### 3.1.2. Changes in Rheology and Pore Size Distribution

Th effect of HbQF on cell lysis was examined in terms of the change in the rheological features of the sludge, showing a shear thinning behavior after treatment. Rheology is the study to quantitatively investigate how a material will deform as a function of force, time, and spatial orientation [28]. In this analysis, with increasing shear stress, the change in shear rate was measured, and the viscosity was calculated based on this.

**Figure 7:**
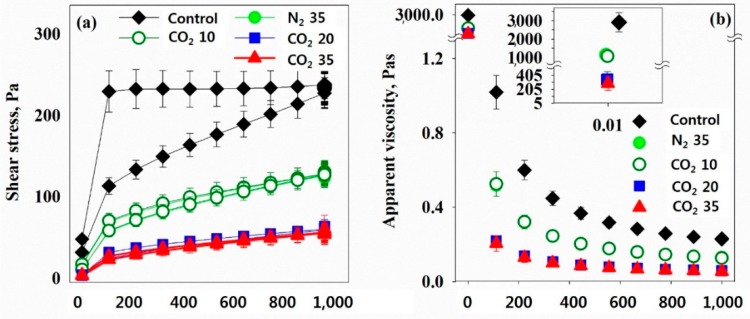
Hysteresis loop and viscosity of various sludge samples; (**a**) relationship between the applied stress and shear rate response of the control and the HbQF-treated sludge under various test conditions; (**b**) viscosity of the sludge.

Figure 7a shows the relationship between the applied stress and shear rate response of the control and the HbQF-treated sludge under various test conditions, and showing non-Newtonian behavior. The control sludge sample showed higher stress values along the ascending path compared to the descending path, resulting in a hysteresis loop between the ascending and descending paths. The loop area is a parameter showing the strength of the thixotropic properties. Increasing the degree of HbQF, the shear stress response at a given shear rate decreases and the loop area does as well, which results in a decrease in the viscosity of the sludge (Figure 7b). This is because of the destruction of sludge flocs or the lysis of microbial cells in the sludge samples following the HbQF treatment.

**Figure 8:**
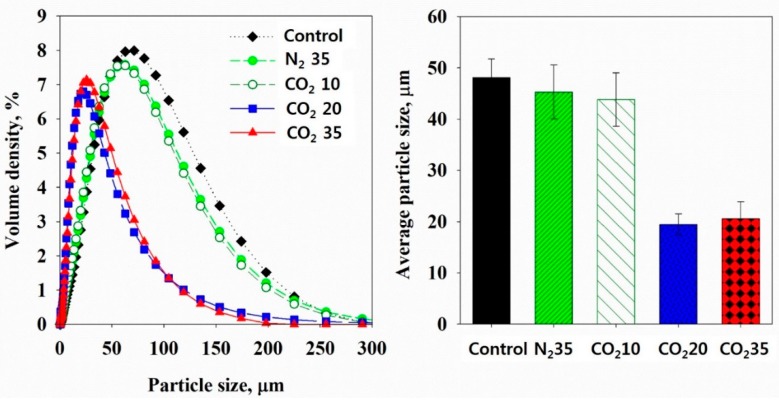
Particle size distribution (**left**) and average particle size (**right**) of sludge in the μm range.

Particle size distribution was measured in the μm and nm ranges. Figure 8 shows the distribution and average of the sludge particles in the μm range. The control sample showed a particle size distribution within the largest size range and an average particle size of 48.1 (±3.6) μm. The N_2_ at 35 bar and CO_2_ at 10 bar samples showed a particle size distribution in a smaller size range than that of the control and the average particle sizes were 45.3 (±5.2) μm for N_2_ at 35 bar and 43.8 (±5.2) μm for CO_2_ at 10 bar. This was a result of the stirring, pressure swing, and CO_2_ dissolution slightly disintegrating the sludge flocs. The CO_2_ at 20 bar sample had a particle size distribution in a significantly smaller size range and the average particle size was 19.5 (±2.0) μm. In addition, the CO_2_ at 35 bar samples had a particle size distribution in a similar size range of that of the CO_2_ at 20 bar. Through the sludge particle size distribution in the μm range, it was determined that HbQF significantly disintegrated sludge flocs.

**Figure 9:**
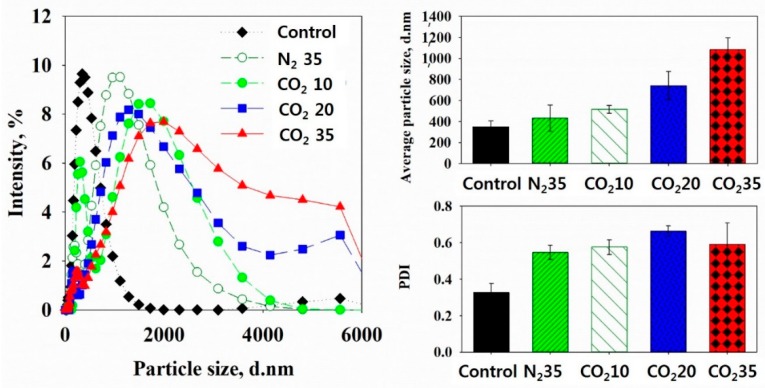
Particle size distribution, average particle size, and polydisperse index (PDI) in the nm range.

To measure the particle size distribution of particles smaller than 0.8 μm, the particle size distribution in the nm range was measured. Figure 9 shows the particle size distribution, average particle size, and polydispersity index (PDI) of the sludge samples. The PDI is the degree of graph dispersity. If the PDI increases, the particle sizes present in the sample are more varied; however, if the PDI decreases, the particle sizes present in the sample are less varied.

The control had only a single peak in the less than 1000-nm range. Sludge in the N_2_ at 35 bar and CO_2_ at 10 bar samples showed two peaks including a small peak in the less-than-1000-nm range and a large peak in the less-than-4000-nm range. The peak in the less-than-1000-nm can be considered a result of the originally present fine particles. In addition, the other peak at less than 4000 nm can be considered a result of the fine particles produced by the slight sludge disintegration. Considering the particle size distribution in the μm range, as the sludge was disintegrated via stirring, the pressure swing, and CO_2_ dissolution, the sludge was divided into fine particles in the nm range. Via this slight disintegration, sludge of the N_2_ at 35 bar and CO_2_ at 10 bar samples had two peaks in the nm-range particle size distribution. Sludge of the CO_2_ at 20 bar and CO_2_ at 35 bar samples showed three peaks including the smallest peak at less than 1000 nm, the largest peak at less than 4000 nm, and another peak at greater than 4000 nm. The last peak above 4000 nm was also likely a result of the fracturing of lysed cells in the um range.

The average particle size of the control was the smallest, 350 (±57) nm, and its PDI was 0.327 (±0.0496). The average particle sizes were 432 (±124) nm for the N_2_ 35 at bar and 516 (±37) nm for the CO_2_ at 10 bar samples. Their PDIs were 0.547 (±0.0389) and 0.57 (±0.0403), respectively. Because of the slight sludge disintegration as a result of stirring, the pressure swing, and CO_2_ dissolution, the average particle sizes for the N_2_ at 35 bar and CO_2_ at 10 bar were greater than in the control, and the PDIs also increased. The average particle size was 741 (±135) nm in the CO_2_ at 20 bar and 1084 (±112) nm in the CO_2_ at 35 bar samples, and their PDIs were 0.663 (±0.0283) and 0.591(±0.1160), respectively. The average particle sizes in the CO_2_ at 20 bar and CO_2_ at 35 bar samples were greater than those in the control, N_2_ at 35 bar, and CO_2_ at 10 bar samples. As more sludge flocs disintegrated via HbQF and more fine particles were present in the sample, the average particle size and PDI increased. The HbQF could disintegrate more sludge flocs and produce a large number of fine particles of a much more diverse size.

#### 3.1.3. Capillary Suction Time, Time to Filter, and Filtration Resistance

**Figure 10:**
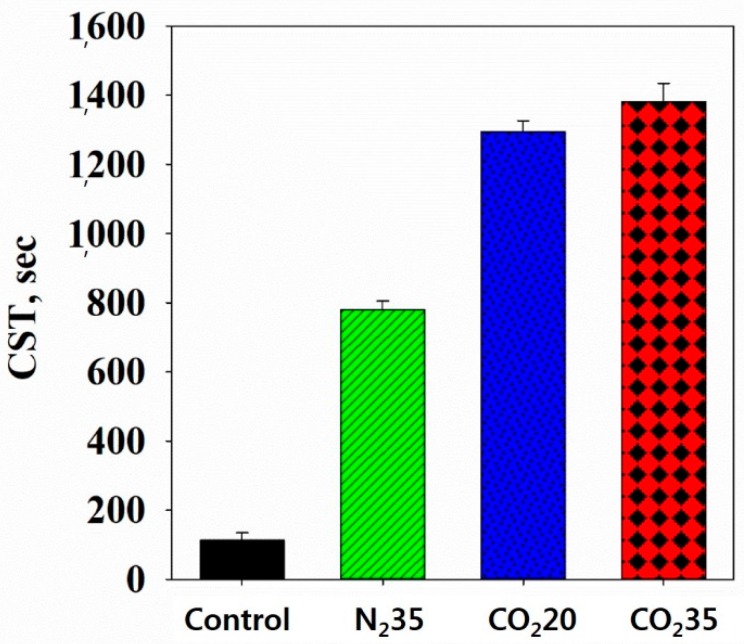
CST of sludge samples.

After cell lysis by HbQF, CST, TTF, and filtration resistance were analyzed to investigate the dewatering efficiency of the HbQF treatment when combined with filtration-based post-treatment. CST is the time for the liquid separated from sludge to pass from one point of a filter to another point. As CST increases, the liquid takes more time to pass through the filter and the sludge has a higher binding strength between the water and sludge floc. Figure 10 shows that the CST of the N_2_ at 35 bar (780.23 (±24.850) s) sample was higher than that of the control (113.17 (±21.942) s), and the CST of the CO_2_ at 20 bar sample (1294.27 (±31.986) s) was higher than that of the control and N_2_ at 35 bar sample. The CST of the CO_2_ at 35 bar sample (1381.10 (±53.767) s) was slightly higher than that of the CO_2_ at 20 bar sample. This result was because of the sludge disintegration [29]. As the sludge disintegrated, organic materials and fine particles released, preventing the liquid from flowing through the sludge and filter. The fine particles disintegrated from the sludge also blocked the filter pores and prevented the filtrate from passing through the filter pores.

**Figure 11:**
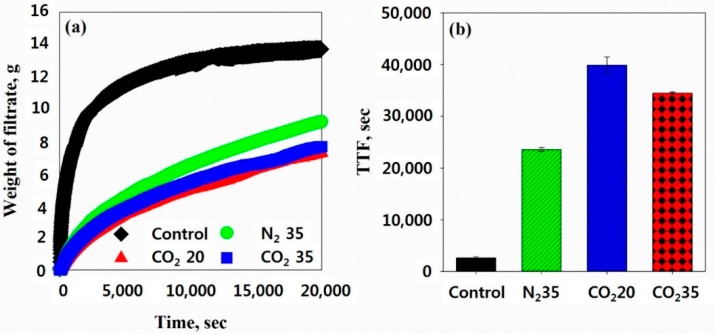
(**a**) Weight of the filtrate during the filtering test and (**b**) TTF (time at which the weight of the filtrate reaches one-half the weight of the initial sample)

The filtering test and TTF were conducted to measure the sludge filtering rate. The faster the filtrate is separated, the higher the sludge filtering rate. Figure 11a shows the weight of the filtrate increased with filtering time for all cases. However, the filtrate of the N_2_ at 35 bar sample was slower than the control. In addition, the filtrates of the CO_2_ at 20 bar and CO_2_ at 35 bar samples separated much more slowly than the others. These results show HbQF slows sludge filtration because of the resistances caused by the lysed cells.

Y. Liu, et al. (2003) [26] determined the relationship between the number of soluble organic materials and the sludge dewatering effect with respect to the filter-based dewatering process. When the number of soluble organic materials increases to a certain amount, the organic materials result in a more flocculated sludge and the dewatering effect increases. However, if there is a much greater number of soluble organic materials, the sludge is increasingly hydrophilic, and the sludge dewatering effect decreases. Despite the increase in dewatering efficiency via the cell lysis and the separation of the inner water from the lysed cells, the index of the CST and TTF showed the dewatering efficiency decreased using the HbQF treatment. This is because the CST and TTF are indexes determining the dewatering efficiency based on filtration.

**Figure 12:**
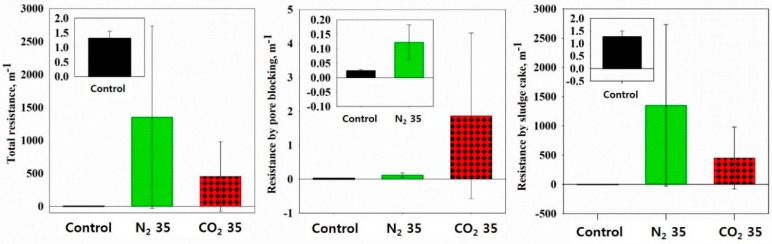
Total resistance, resistance by sludge cake, and pore blocking.

To determine the major factor causing resistance during the sludge filtering test, the resistance analysis was numerically compared. Figure 12 shows the causes of the permeation resistance when each sludge sample passes through the filter. Total resistance of the control was much smaller than other cases, and the total resistance in the N_2_ at 35 bar sample was significantly greater than that of the CO_2_ at 35 bar sample. The resistance via pore blocking in the control and the N_2_ at 35 bar samples was small and negligible, respectively, but the resistance via pore blocking in the CO_2_ at 35 bar sample showed slightly higher values. This was because through the treatment under CO_2_ at 35 bar, more sludge flocs disintegrated and more fine particles formed. These fine particles were blocked during the filtering test and inhibited the filtrate from flowing through the filter pores. In all cases, the resistance by the membrane itself was negligible and the resistance by the sludge cake was nearly that of the total resistance. This shows the major factor that deteriorated the sludge dewaterability using the filtration-based method was not the clogging of the fine particles but the sludge cake deposition on the filter. The increased resistance after cell lysis agreed with previous studies [22,30].

### 3.2. Effect on Dewaterability and the Release of Sludge Organic Materials.

The dewatering efficiency of the HbQF combined with settling post-treatment was investigated. In this study, the volume of the separated liquid and solid precipitated during settlement were compared.

**Figure 13:**
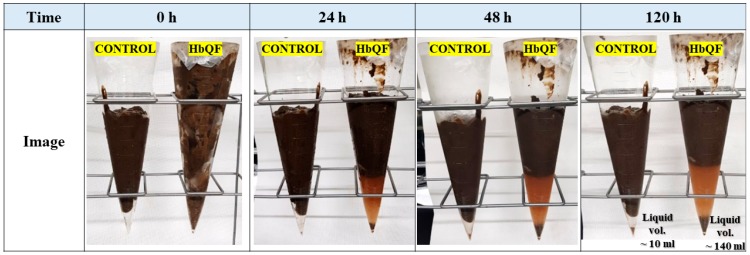
Images of sludge samples according to the time thawed in a refrigerator.

Figure 13 shows the sludge sediment behavior over time while maintaining and thawing the sludge samples in a refrigerator. In the case of the control sample, it can be seen that the solid–liquid separation did not work well regardless of the thawing time in the refrigerator. However, in the case of the HbQF treated sample, the solid–liquid separation was apparent in the images after 24 h. The liquid volume of the control was approximately 10 mL after 120 h, but 140 mL for the HbQF-treated sample (CO_2_ 20 bar). This shows the HbQF treatment disintegrated sludge flocs and fine particles were more dispersed in the supernatant; the final supernatant volume was much greater in the HbQF-treated sample. The supernatant containing high organic compounds can also be utilized for cultivation in other biological processes of treatment plants.

Organic materials in the sludge samples were separated through a chemical process such as alum coagulation, and physical processes, such as centrifugation and ultrasonification. Each sample prepared using the method shown in Figure 3 was termed (a) Alum, (b) Centrifuge 5000× *g*, (c) 1st Ultrasonication, and (d) 2nd Ultrasonication. The order of separation of the organic matter increased in the order of Alum, Centrifuge 5000× *g*, 1st Ultrasonication, and 2nd Ultrasonication. The organic material detected in the sample of Alum is that which was weakly bonded to the inside of the sludge; however, the organic material of the 2nd Ultrasonication is that which was released after agglomeration, centrifugation, and two additional ultrasonic treatments.

**Figure 14:**
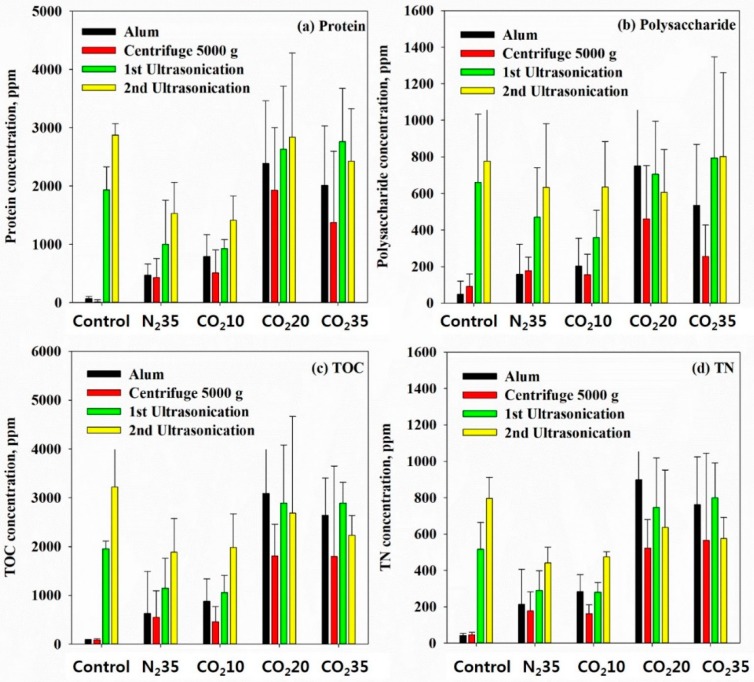
Distribution of organic compounds in the concentrated sludge: (**a**) protein; (**b**) polysaccharide; (**c**) TOC (total organic carbon); (**d**) TN (total organic nitrogen).

Concentrations of protein, polysaccharide, total organic carbon, and total nitrogen were measured (Figure 14). In the case of the protein concentration of the control, the Alum and Centrifuge 5000× *g* showed a negligible amount. According to J. Kopp et al. [6], centrifugation cannot release strongly bound organic materials. That is, the organic materials detected in Alum and Centrifuge 5000× *g* can be considered organic materials that were originally positioned outside the sludge flocs, and there was a very small amount of protein dispersed out of the sludge flocs. The N_2_ at 35 bar and CO_2_ at 10 bar treatments show the concentration of protein increased. As can be seen from the particle size distribution in Figure 8, the sludge flocs and cells of the N_2_ at 35 bar and CO_2_ at 10 bar samples were slightly disintegrated and fractured by the mechanical mixing in a pressurized reactor, increasing the concentration of the protein compared to the control. However, the HbQF treatment significantly broke the cells and caused the inner proteins to flow out, increasing the protein concentration of the leachate.

During the first and second ultrasonic stages, which can release tightly bound organic matter, a large amount of organic matter was measured in the control, and a slightly reduced amount of protein was measured in the N_2_ at 35 bar and CO_2_ at 10 bar samples. This is because during the mixing of these samples, some of the broken sludge floc and cells were removed during sampling of Alum and Centrifuge 5000× *g*, and the amount of protein reduced by the removed moiety was detected in the subsequent ultrasonic treatment. However, in the case of the CO_2_ at 20 bar and CO_2_ at 35 bar samples which did undergo a rapid-freezing process, the amount of organic matter measured during the first and second ultrasonic steps was similar to that of the control. This seems to be because most of the sludge particles broken into fine particles by HbQF were further broken down finely by the ultrasonic waves, passing through the 0.45-μm filter used for sample preparation and increasing the concentration of proteins in the samples of the 1st Ultrasonication and 2nd Ultrasonication. Polysaccharide, total organic carbon, and total nitrogen concentrations showed the same trend as the protein concentration.

## 4. Conclusions

In this study, the effect of HbQF on cell rupture and sludge dewatering was investigated. CO_2_ gas hydrates formed under a condition of a 0.2 (±0.1) °C temperature and a greater than 20 bar pressure in the sludge sample; the sludge rapidly froze as the pressure was released to atmospheric pressure. The behavior of the temperature change under decompression of the sludge samples could be classified into three classes: (i) control, (ii) N_2_ 35 bar and CO_2_ 10 bar, (iii) CO_2_ 20 bar and CO_2_ 35 bar. As the microbial cells ruptured because of HbQF, organic materials were released from the cell and the soluble organic material concentration increased. The cell rupture increased the conductivity of the supernatant from 10 mS/cm to 14 mS/cm and the osmotic pressure from 122.8 mmol/kg to 162.3 mmol/kg. HbQF also changed the surface charge of sludge samples to a more negative value (from −13.10 mV to −16.63 mV) via the released organic materials. In the rheological study, it was found that the inner structure of the sludge including the microbial cells was broken down and both the area of the hysteresis loop and viscosity decreased. A dewatering test based on filtration methods showed the ruptured microbial cells resulted in a more compact sludge cake and finely lysed particles blocked the filter pores. The resistance by the sludge cake was the major factor that decreased the sludge dewatering effect when filtration was used. However, as the inner substance which was originally positioned in the cell was released by HbQF, the precipitated solid volume in the settling test decreased, alleviating the treatment loading. The supernatant of HbQF treated samples was organic rich and could be further used for organic cultivation. This study is the first using gas hydrate dissociation energy in a treatment aimed at reducing sludge, and shows HbQF with settling post-treatment improves cell rupture and sludge dewaterability along with partial disinfection of sludge samples.

(a) Dissociation of CO_2_ gas hydrates rapidly freezes sewage sludge.
(b) HbQF releases organic materials from sludge floc and microbial cells.
(c) HbQF reduces cell viability by cell lysis.
(d) HbQF followed by gravitational settling could be the solution to dewater sludge.

## Figures and Tables

**Table 1:** Nomenclature and reaction condition of each sample.

Sample Name	Control	N_2_ 35	CO_2_ 10	CO_2_ 20	CO_2_ 35
Pressure, bar	-	35	10	20	35
Guest molecule	-	N_2_	CO_2_	CO_2_	CO_2_
Temperature, °C	-	0.2	0.2	0.2	0.2
Stirring rate, rpm	-	150	150	150	150

**Table 2:** Zeta potential of sludge particles treated by each HbQF treatment.

Sample	Raw	Control	N_2_ 35	CO_2_ 10	CO_2_ 20	CO_2_ 35
**Average, mV**	−13.10	−11.12	−15.40	−15.30	−16.63	−21.00
**Standard deviation, mV**	1.127	0.743	0.400	1.916	0.833	2.258
